# Protocol for a single-centre, parallel-arm, double-blind randomised trial evaluating the effects of tourniquet use in total knee arthroplasty on intra-operative and post-operative outcomes

**DOI:** 10.1186/s12891-018-2352-8

**Published:** 2018-12-06

**Authors:** Richard S. Page, Simon Williams, Avanthi Selvaratnam, Shaun Waring, Myles Conroy, Andrew Thomson, Sally Beattie, Rekha Ganeshalingam, Stephen D. Gill

**Affiliations:** 1 Barwon Centre for Orthopaedic Research and Education (B-CORE), Barwon Health and St John of God Hospital Geelong, 80 Myers St, Geelong, 3220 Australia; 2 School of Medicine, Deakin University, 75 Pigdons Road, Waurn Ponds, 3216 Australia; 3 Orthopaedic Department, University Hospital Geelong, 272-322 Ryrie Street, Geelong, 3220 Australia; 4 Anaesthetics Department, University Hospital Geelong, 272-322 Ryrie Street, Geelong, 3220 Australia

**Keywords:** Knee arthroplasty, Tourniquet, Knee pain, Quadriceps strength

## Abstract

**Background:**

Tourniquet use during total knee replacement is common, yet uncertainty exists regarding its benefits and harms. The primary aim of the current study is to investigate whether tourniquet use during total knee replacement leads to greater reduction in quadriceps strength than non-tourniquet use at three months post-surgery. Secondary aims include investigating the effects of tourniquet use on: quadriceps strength at day 2 and 5, and 12 months post-surgery; pain and analgesia requirements; self-reported physical function and quality of life; blood loss and replacement; surgeon satisfaction with the intra-operative visual field; operation and anaesthetic time; complications; cement mantle quality; patient satisfaction; and hospital length of stay.

**Methods:**

The study is a single centre, parallel-arm, double-blind (participant and assessor), randomised trial with 1:1 random allocation. Participants will be undergo total knee replacement with or without tourniquet. Linear mixed models will be used for group comparisons of continuous outcomes available at multiple timepoints. Other continuous outcomes that are assessed at baseline and once/twice at follow-up will be analysed using linear regression. Categorical outcomes will be analysed using logistic regression models.

**Discussion:**

This study will provide high-quality evidence regarding the effects of tourniquet use during total knee replacement, which can be used to inform surgeon decision-making.

**Trial registration:**

Australian New Zealand Clinical Trials Registry ACTRN12618000425291. Retrospectively registered 23 March 2018.

## Background

Total knee replacement (TKR) is a common and successful procedure, with over 1 million TKRs occurring annually in OECD countries [1]. TKR is regularly performed using a tourniquet, with usage in 37–93% of surgeries [2, 3]. However, tourniquet use during TKR is debated due to evidence questioning the advantages, and the possibility of increased complications [4, 5].

A thigh tourniquet compresses the leg and restricts distal blood flow which is intended to reduce intra-operative blood loss at the surgical site. Tourniquet use has been suggested to improve surgical field view, allow cement to bond more effectively [6], and produce shorter operating time which might reduce the risk of infection [4]. A systematic review found tourniquet use reduced intra-operative blood loss (198 ml) and operating time (5 min), but did not affect post-operative blood loss or the possibility of requiring transfusion [4]. Tourniquet use increased the risk of thrombotic events such as deep vein thrombosis (DVT) and pulmonary embolism (PE) (risk ratio (RR) 5.00; 95% CI, 1.31 to 19.10), and non-thrombotic complications such as reoperation, haematoma, or nerve palsy (RR, 2.03; 95% CI, 1.12 to 3.67). Knee range of movement in the first 10 days post-operatively was 10.4 degrees less in the tourniquet group. More recently, Rathod et al. [7] found no difference in cement penetration when a tourniquet was used from incision to arthrotomy closure compared to using a tourniquet only during cementation. Pfitzner et al. [8] found cement mantle thickness was 1.2 mm (*p* = .009) greater in the tourniquet group than non-tourniquet group, and Ledin et al. [9] found no difference in prosthesis migration in tourniquet versus non-tourniquet TKR. Several investigators have reported higher post-operative pain when a tourniquet was used compared to no tourniquet [9–11].

Quadriceps function influences post-operative physical performance, functional ability and rehabilitation following TKR [12, 13]. Quadriceps dysfunction following TKR can be immediate, profound and persist for years after surgery, resulting in substantial functional deficits [14]. Mizner et al. [15] found quadriceps strength was 62% less than pre-operative values when measured four weeks after TKR.

Tourniquet use during TKR has been implicated in quadriceps dysfunction. Two studies, both with small samples (*n* = 20 & 28) assessed muscle function following TKR. Liu et al. [16] found that tourniquet patients had significantly less quadriceps muscle activity on EMG for the first six months post-operatively, as well as increased pain on day two and four post-operatively compared to non-tourniquet patients. Dennis et al. [17] found tourniquet patients had less isometric quadriceps strength when assessed with a force transducer at three weeks and three months post TKR compared to non-tourniquet patients.

The mechanism to explain quadriceps dysfunction following TKR and tourniquet use is unclear. A commonly accepted pathway is that ischaemia induces acute inflammation, degeneration and necrosis of muscle fibres [18]. Muscle biopsy following anterior cruciate ligament surgery with tourniquet found an accumulation of lysosomes, edema of fibres and endothelium, and fibre necrosis [18]. Tourniquet use might also injure nerves and/or delay nerve conduction and muscle activation. Mizner et al. [15] investigated quadriceps strength after TKR and found loss of strength was largely explained by a combination of reduced voluntary muscle activation and atrophy, but muscle activation played a greater role. Interestingly, most activation failure seemed unrelated to knee pain during muscle contraction, contrary to suggestions of pain-induced muscle inhibition.

### Objectives

The primary objective of this study is to determine whether non-tourniquet use during TKR reduces quadriceps strength less than tourniquet use when measured three months post-operatively.

A secondary objective is to determine whether non-tourniquet use during TKR reduces quadriceps strength less than tourniquet use at day 2 and 5, and 12 months post-operatively.

Other secondary objectives are to determine the effects of tourniquet use on:

1. Pain and analgesia requirements
2. Self-reported physical function and quality of life
3. Blood loss and replacement
4. Surgeon satisfaction with the intra-operative visual field
5. Operation and anaesthetic time
6. Complications including revision surgery
7. Cement mantle quality
8. Patient satisfaction
9. Hospital length of stay

## Methods

### Study design

The study is a single centre, parallel-arm, double-blinded (participant and assessor), randomised trial with 1:1 random allocation. The study schedule is summarized in Table 1.

**Table 1:** Study schedule

	Pre-randomisation	Surgery Day 0	Post-surgery				
			Day 2	Day 5	During inpatient stay	3 months	12 months
Enrolment							
Eligibility screen	X						
Informed consent	X						
Randomisation		X					
Interventions							
Tourniquet TKR		X					
No tourniquet TKR		X					
Assessments							
Demographic variables	X						
Quadriceps strength	X		X	X		X	X
Blood loss and replacement		X			X		
Surgeon satisfaction		X					
Operation and anaesthetic time		X					
Tourniquet inflation time		X					
Pain			X	X			
Morphine equivalent daily dose					X		
Complications					X		
Knee Society Score	X					X	
Oxford Knee Score	X					X	X
WOMAC	X					X	X
EQ-5D-5 L	X					X	X
Revision surgery							X
Cement mantle							X

### Setting

The study will be conducted at a large regional public health service in Victoria, Australia. Twelve surgeons perform TKRs at the centre, all of whom will be involved in the study. In 2014, 149 primary TKRs were completed at the centre.

### Eligibility criteria

Eligible participants must have the following characteristics:

1. Undergoing primary TKR for primary osteoarthritis
2. ≥ 18 years of age
3. Willing, able and mentally competent to provide informed consent (able to read and understand the Patient Information and Consent Form which is written in English language).

People who have the following pre-operative characteristics are not eligible:

1. Undergoing bilateral TKR (as participant characteristics and rehabilitation are different to unilateral TKR)
2. Neurological deficit affecting the surgical knee (due to potential effects on quadriceps strength)
3. Rheumatoid arthritis (different aetiology than osteoarthritis)
4. Pre-operative knee flexion < 60° (degree of flexion required for strength testing)
5. Varus/valgus deformity > 15° (requires different surgical approach)
6. Opioid tolerant (current use of oxycontin, opioid patches, or tramadol; > 4 tabs panadeine forte per day) (unable to assume standardised analgesia pathway)
7. Sulphonamide allergy (to allow parecoxib/celecoxib use)
8. Intolerant/allergic to oxycodone (unable to assume standardised analgesia pathway)
9. Poorly controlled diabetes (HbA1C > 8) (impacts on choice of dexamethasone as antiemetic)
10. Cognitively impaired (mini-mental state examination of < 25/30 [19]) (affects consent and participation in rehabilitation)
11. eGFR < 60 mL/min/1.73m^2^ (allows parecoxib/celecoxib use)
12. Privately insured patients (unable to follow-up)

All patients attending the study-site for pre-operative assessment for TKR will be assessed for eligibility by the surgeon, orthopaedic registrar or research coordinator. Eligible participants will be invited to participate in the study and informed written consent obtained as appropriate. Participation in the study is voluntary; no financial incentives will be offered.

Considering the expected number of participants fulfilling inclusion and exclusion criteria at the study site, recruitment is expected to occur over a 4-year period, commencing in October 2014.

### Randomisation

People who meet eligibility requirements and provide informed consent will be randomly allocated to either tourniquet or non-tourniquet groups with a 1:1 allocation ratio in blocks of 10. The allocation sequence will be computer generated by the research coordinator prior to trial commencement. Allocation will be concealed until immediately prior to anaesthetic induction, at which time the surgeon will access the allocation code for that participant via an opaque sealed envelope.

### Blinding

All participants, clinical staff and research staff will be blinded to group allocation, with the exception of the treating surgeon/s and theatre staff.

### Surgery

One of 12 surgeons will complete each TKR, with training registrars operating under direct supervision. Prosthesis type and whether to use navigated or non-navigated TKR is at the surgeon’s discretion, which will be decided upon prior to knowledge of the participant’s group allocation. A medial parapatella approach and no drain will be used for all participants.

The tourniquet group will have a tourniquet applied with padding. After exsanguination of the operated limb using a rubber tube or esmarch exsanguinator, the tourniquet will be inflated to 100 mmHg above systolic blood pressure or 250 mmHg, whichever is higher. The tourniquet will be deflated immediately prior to wound closure.

All participants receive intravenous Tranexamic Acid (TXA) to reduce peri-operative bleeding. The typical dose is 1 g TXA diluted in 100 ml normal saline infused intravenously at induction. Once the participant is in the recovery room, a second dose of 1 g TXA in 100 ml normal saline is given via infusion pump over 8 h (12.5 ml/hr).

All participants receive DVT prophylaxis commencing six hours after TKR unless contraindicated: clexane 40 mg daily for 14 days. Mechanical DVT prophylaxis via foot pumps will be applied until the patient commences ambulating at least 5 m daily.

### Anaesthesia, pain management and transfusion

Anaesthesia and analgesia are according to the organisation’s standardized protocols. All participants receive general anaesthesia with inhaled sevoflurane. Post-operative analgesia includes sub-sartorius saphenous nerve catheter infusion with patient controlled boluses, and paracetamol, celecoxib and oxycontin. Oxycodone is given for breakthrough pain. If a participant reports severe posterior knee pain that is unresponsive to first-line analgesia, a single sciatic nerve block will be considered.

Blood transfusion will occur if 1) the participant’s haemoglobin is less than 80, or less than 100 for patients with a history of significant cardiac pathology such as ischaemic heart disease or 2) the participant is hypotensive (i.e. systolic blood pressure < 100 mmHg and associated tachycardia) with suspected hypovolemia that is unresponsive to crystalloid/colloid fluid replacement.

### Post-operative care and rehabilitation

Post-operative care of all participants, irrespective of group allocation will be according to the organisation’s TKR protocols and care pathways. Participants are mobilized day-one post-operatively and participate in a daily rehabilitation program as coordinated by Allied Health staff. Participants are discharged to their usual place of residence once they are medically fit and sufficiently independent with activities of daily living. Participants are sent to inpatient rehabilitation if they are not sufficiently independent to manage at home, which often coincides with living alone. Following discharge from inpatient care, all participants receive ongoing rehabilitation under the direction of Allied Health staff, which is ceased at the discretion of staff and participants. The organisation’s care pathways allow professional discretion regarding the amount and content of rehabilitation completed. Complete standardization of each group’s rehabilitation program is beyond the jurisdiction of the current study and is a potential limitation. Participation in ongoing rehabilitation will be recorded, equivalence between groups assessed and differences will inform data analysis and interpretation.

### Outcome measures and assessment time points

The primary outcome is the maximum percentage change in isometric quadriceps strength assessed preoperatively and 3 months following TKR. Strength will be measured in Newtons and assessed using a fixed-base electromechanical dynamometer (IsoForceControl EVO2 dynamometer [20]) with the knee stabilised in 60 degrees of flexion. Patient will be seated in a customised chair with a frame that fixes the dynamometer in position. The force plate will be applied adjacent to the malleoli of the ankle. Following 1–2 practices, participants will extend their knee as forcefully as they can for 10 s. The maximum force from three consecutive attempts will be recorded. Fixed-based dynamometer has very good to excellent reliability in people following arthroplasty [21].

Quadriceps strength at day 2 and 5, and 12 months post-operatively are secondary outcomes. Other secondary outcomes include:

1. Post-operative inpatient pain and analgesia requirements
  - Knee pain intensity on day 2 and 5 post-operatively according to a 0–10 numeric scale (0 = no pain, 10 = extreme pain)
  - Morphine equivalent daily dose [22] (mg, average for first 5 days)
2. Blood loss and replacement
  - Intra-operative blood loss (ml, sucker bottle minus irrigation volume)
  - Transfusion (units)
3. Surgeon satisfaction with intra-operative visual field
  - 1–10 numeric scale (1 = completely unsatisfied, 10 = completely satisfied)
4. Operation and anaesthetic time (minutes)
5. Complications during inpatient stay
  - DVT or PE
  - Wound complications such as infection, haematoma or breakdown which require a change in management such as antibiotics or reoperation
  - Medical complications (Medical Emergency Team (MET) calls [23] or death)
6. Hospital length of stay (days)
7. Self-reported pain, physical function and quality of life at 3 and 12 months
  - Knee Society Score (KSS) [24]
  - Oxford Knee Score (OKS) [25]
  - WOMAC [26]
  - EQ-5D-5 L [27]
8. Revision surgery within 12 months
9. Cement mantle quality at 12 months [28]
10. Patient satisfaction at 3 and 12 months

Strength measurements will be collected by research assistants, who are trained by a study investigator. The KSS will be completed by the treating surgeon, training registrar or resident. Participants will complete standardised questionnaires in paper-format with assistance offered by a research assistant as required. Cement mantle quality will be determined by a surgeon or research assistant trained by a surgeon. Data for the remaining outcomes will be extracted from the participant’s medical record. Research assistants will enter data into REDCap, the study’s password-protected electronic data collection and management tool hosted by the institution [29].

The study will collect baseline demographic information including age, sex, height, body weight, American Society of Anesthesiologists (ASA) score [30], cognitive function (mini-mental state examination [19]) and medical comorbidities summarized with the Charlson Comorbidity Index [31]. Prosthesis type and the use of navigated or non-navigated procedures will be recorded.

Once participants are enrolled in the study and undergone surgery, every reasonable effort will be made to reassess them for the entire study period. Research assistants will attempt to contact participants a maximum of four times over a three-month period using phone, email or mail before they are considered lost to follow-up. Participants may withdraw from the study at any time and for any reason. Participants will be invited, though not required, to indicate reasons for withdrawal. Those wishing to withdraw from the study will be invited to complete questionnaire assessments via mail rather than attending reassessment/s in person.

### Adverse events and data safety and monitoring

An adverse event refers to an untoward occurrence during the study, which may or may not be causally related to the intervention [32]. We will collect information relating to adverse events from randomisation until the participant completes the 12 month post-operative assessment.

Serious adverse events (SAE) are those which result in death, are immediately life-threatening, rehospitalisation, result in persistent or significant disability or incapacity, or have important clinical sequelae. Serious adverse events will be reported to the organisation’s Human Research Ethics Committee. All adverse events will be reviewed on a monthly basis by senior surgeons in the organisation’s orthopaedic department. Senior surgeons will consider the likely contribution of tourniquet use towards each complication and recommend to the investigators whether to modify or cease the study based on their findings. The surgeon whose patient had the adverse event will be excluded from the final decision making regarding whether the event is related to tourniquet use. Annual reports of the study’s progress will be sent to the organisation’s Human Research Ethics Committee.

### Statistical analysis plan

The main results will be based on intention-to-treat analysis which will include all participants as randomised. Per protocol analysis will also be conducted as secondary analysis and include only patients whose surgery was completed as randomised. All categorical data will be summarised using frequencies and percentages and baseline characteristics will be compared using the Chi-squared statistic. Interval or continuous data will be summarised using means with standard deviations or medians with lower and upper quartiles if the data are skewed. The amount of missing data for each group and each outcome will be described with frequencies and proportions. Analysis will include cases with available data. No imputation of missing data will occur. For questionnaire data, if a participant has not responded to ≥15% of questions in a questionnaire (or subscale where relevant), the responses for that scale will not be included in the analysis.

Linear mixed models [33] will be used for group comparisons of quadriceps strength at the different follow-up time points (2 days, 5 days, 3 months and 12 months). The major advantages of using this method are that it accounts for intra-individual correlations in observations, multiple variables can be included in the model and the method uses all available data even in the presence of unbalanced data. If assumptions permit, the restricted maximum likelihood approach will be adopted. The models will include an interaction term between time and treatment group, which will indicate the between group differences in quadriceps strength changes from baseline. The linear mixed model will also be considered for the analysis of continuous secondary outcomes that are available at multiple follow-up time points (> 3 time points). Other continuous outcomes that are only assessed at baseline and once/twice at follow-up will be analysed using linear regression, allowing for estimation of clustered sandwich error estimates [34]. Non-parametric models such as quantile regression will be considered for cases where assumptions of linear models are not satisfied.

Secondary outcomes that are categorical will be analysed using logistic regression models [35]. Count data such as hospital length of stay will be analysed using Poisson regression or other count-data models (e.g. negative binomial regression) if the assumptions of the Poisson regression models are not satisfied [36].

Questionnaire data will be analysed as a total score for the OKS, or component score for the WOMAC (Pain, Stiffness and Function), KSS (Knee Score and Function Score) and EQ-5D-5 L (descriptive system and VAS).

To explore the relationship between quadriceps strength and patient function, strength will be correlated with patient reported outcomes using Pearson’s correlation coefficient [37].

Relevant tests will be two-sided and considered significant if *p* values are less than 0.05. Stata Statistical Software version 14 or later or R Statistical Packages version 3 or higher will be used for analysis.

### Sample size

The sample size was calculated on the basis of the primary outcome. To the best of the our knowledge, at the time of study development there was no published data reporting quadriceps strength following tourniquet use and total knee replacement that could be used to estimate a sample size for this study. Therefore, allowing for a medium to large effect size (Cohen’s d = 0.65), based on the large quadriceps function differences between groups observed by Liu et al. [16] which were measured using surface electromyography, a two-sided significance level of α = .05 and power of 80%, a minimum sample size of 39 participants per arm was estimated. Allowing for a 15% drop-out rate, we aimed to recruit 45 participants to each group.

### Ethics and dissemination

Barwon Health Human Research Ethics Committee, Geelong, Australia approved the study including the protocol and the participant information and consent form (reference 11/89). The Ethics Committee will be notified of any adverse events relating to the study or any changes to the study protocol. The study complies with the National Statement on Ethical Conduct in Research [38]. The study is registered with the Australian New Zealand Clinical Trials Registry (ref: ACTRN12618000425291) [39].

All investigators and the trial statistician will have access to the final dataset. Key study results will be shared with interested participants in writing using plain English. Results will be disseminated at national and international conferences and in peer-reviewed journals. Authorship eligibility for disseminated material will be determined according to international criteria [40].

## Discussion

Th current study will fill a knowledge gap and provide much needed empirical evidence regarding the effects of tourniquet use in TKR. The study results will assist orthopaedic surgeons when deciding on the most beneficial surgical technique for their patients.
